# A Neurobiological Hypothesis of Treatment-Resistant Depression – Mechanisms for Selective Serotonin Reuptake Inhibitor Non-Efficacy

**DOI:** 10.3389/fnbeh.2014.00189

**Published:** 2014-05-20

**Authors:** Jeremy D. Coplan, Srinath Gopinath, Chadi G. Abdallah, Benjamin R. Berry

**Affiliations:** ^1^Division of Neuropsychopharmacology, Department of Psychiatry and Behavioral Science, State University of New York Downstate Medical Center, Brooklyn, NY, USA; ^2^Department of Psychiatry, Yale School of Medicine, New Haven, CT, USA; ^3^Clinical Neuroscience Division, National Center for PTSD, West Haven, CT, USA; ^4^State University of New York Downstate College of Medicine, Brooklyn, NY, USA

**Keywords:** selective serotonin reuptake inhibitors, treatment-resistant depression, glutamate, somatodendritic 5-HT_1A_ autoreceptors, dorsal raphe, hippocampus, lamotrigine, α_2_-heteroreceptors

## Abstract

First-line treatment of major depression includes administration of a selective serotonin reuptake inhibitor (SSRI), yet studies suggest that remission rates following two trials of an SSRI are <50%. The authors examine the putative biological substrates underlying “treatment resistant depression (TRD)” with the goal of elucidating novel rationales to treat TRD. We look at relevant articles from the preclinical and clinical literature combined with clinical exposure to TRD patients. A major focus was to outline pathophysiological mechanisms whereby the serotonin system becomes impervious to the desired enhancement of serotonin neurotransmission by SSRIs. A complementary focus was to dissect neurotransmitter systems, which serve to inhibit the dorsal raphe. We propose, based on a body of translational studies, TRD may not represent a simple serotonin deficit state but rather an excess of midbrain peri-raphe serotonin and subsequent deficit at key fronto-limbic projection sites, with ultimate compromise in serotonin-mediated neuroplasticity. Glutamate, serotonin, noradrenaline, and histamine are activated by stress and exert an inhibitory effect on serotonin outflow, in part by “flooding” 5-HT_1A_ autoreceptors by serotonin itself. Certain factors putatively exacerbate this scenario – presence of the short arm of the serotonin transporter gene, early-life adversity and comorbid bipolar disorder – each of which has been associated with SSRI-treatment resistance. By utilizing an incremental approach, we provide a system for treating the TRD patient based on a strategy of rescuing serotonin neurotransmission from a state of SSRI-induced dorsal raphe stasis. This calls for “stacked” interventions, with an SSRI base, targeting, if necessary, the glutamatergic, serotonergic, noradrenergic, and histaminergic systems, thereby successively eliminating the inhibitory effects each are capable of exerting on serotonin neurons. Future studies are recommended to test this biologically based approach for treatment of TRD.

## Introduction

Treatment resistant depression (TRD) (Souery et al., 2006) is commonly regarded as a failure of depression to respond to two adequate courses of antidepressants (Burrows et al., 1994; Souery et al., 1999). Results from the STAR*D study indicate that <30% of patients with major depressive disorder (MDD) remitted to the selective serotonin reuptake inhibitor (SSRI), citalopram (Trivedi et al., 2006). Remitter rates for the first two steps of STAR*D suggest that close to half of patients with MDD suffered from TRD. We expand on previous reviews of the neurobiology of treatment resistance in depression (Trivedi et al., 2008). Neurobiological-based guidelines as to how to approach this sizeable group of *de facto* TRD patients are discussed. This paper provides a comprehensive review about the serotonin system with limited information about other hypotheses of TRD. We elaborate on the serotonin system and discuss the glutamatergic hypothesis only in the context of serotonin function in TRD briefly. We recognize that we are unable to review other hypotheses of TRD more comprehensively and refer the reader to other sources such as Trivedi et al. (2008) for alternative hypotheses of TRD. We also refer the reader to a paper by Sanacora et al. (2012), which elaborates on the glutamatergic hypothesis of depression. We recognize that our hypothesis may only account for a portion of serotonin pathophysiology in TRD, which may be affected by multiple non-biological factors including social variables. For instance, quality of living environment, as suggested by Branchi (2011), who posits that SSRIs do not elevate mood directly but act as a catalyst by increasing neural plasticity, and therefore accentuate the effects of environment on mood.

Firstly, we formulate a neurobiological hypothesis for TRD based on putative function of the serotonin system. Hypotheses rest in part on serotonin-related functions in panic-anxiety (Coplan et al., 1992, 1995). We had previously examined a paradoxical hyper-vis-à-vis hypo-function of serotonin neurotransmission in panic disorder, which we now extend to mood disorders. Secondly, we utilize specific patterns of SSRI response to delineate a subgroup of patients who demonstrate an *a priori* proclivity toward TRD, using a “pharmacological dissection” approach (Klein, 2008). The patterns may help identification of TRD patients and “short-cut” patients to effective treatment approaches, thus avoiding “serial antidepressant non-response.” Thirdly, we advance the case that TRD responds pharmacologically more like “bipolar” vis-à-vis “unipolar” depression (Angst et al., 2010; Li et al., 2012) although this approach is not without contrary evidence (Healy, 2006; Zimmerman et al., 2008). Finally, we propose a rationale for TRD that invokes the necessity of “stacking” medications, akin to treatment-resistant hypertension or diabetes, balancing the risk of polypharmacy versus continued symptomatology.

## Neurobiology of the Serotonin System in TRD

### Theories for mechanism of SSRI efficacy and potential shortcomings

**Figure 1:**
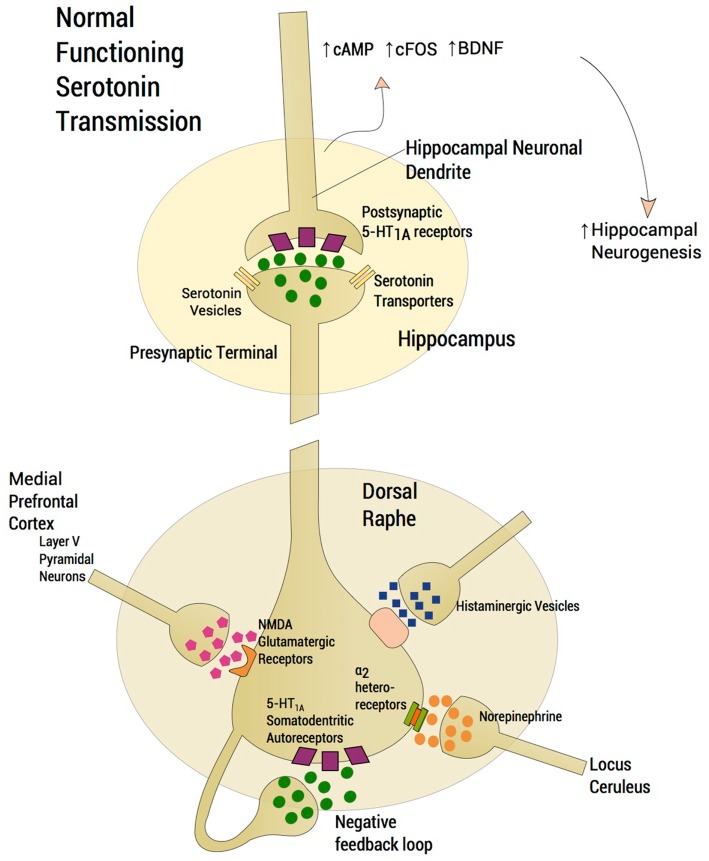
**Normal functioning serotonergic neuron**. The schema depicts a normally functioning serotonin neuron located in the dorsal raphe. A number of features are highlighted. Glutamatergic afferents arise from layer V pyramidal neurons of the medial prefrontal cortex (mPFC), which project, directly via a single axon directly to the dorsal raphe. Activation of 5-HT_2A_ receptors in the mPFC activates these cortical neurons whereas activation of 5-HT_1A_ receptors diminish glutamatergic outflow from the mPFC. The absence of rapid response to SSRIs was attributed to the shutdown of dorsal raphe neuron firing following acute enhancement of a negative feedback loop via somatodendritic 5-HT_1A_ receptors located on the cell bodies of serotonin neurons in the dorsal raphe. Increasing norepinephrine neurotransmission from locus ceruleus firing through α_2_-heteroreceptors produces inhibition of dorsal raphe serotonin neuron firing. Histamine exerts an inhibitory effect on serotonin neuronal firing through histamine H_1_ receptors. Serotonin neurotransmission enhances neuroplastic effects and intracellular transduction cascades following post-synaptic agonism of 5-HT_1A_ receptors located in the hippocampus, ultimately resulting in an increase in neurogenesis. Normative neurotransmission at each synaptic junction is represented by the depiction of five “neurotransmitter molecules” located within the synaptic junction and five in the presynapse.

The serotonin system and SSRIs are a primary area of focus. The reader is referred to other sources (Trivedi et al., 2008) for non-response to other agents. SSRI response has been conceptualized based on seminal studies by Blier and De Montigny (1983); Blier and de Montigny (1987); Artigas et al. (1996). The model (Blier et al., 1998) aimed to understand the mechanism of SSRI action. First, the absence of rapid response to SSRIs was attributed to shut down of dorsal raphe neuronal firing following acute reuptake blockade and enhancement of negative feedback via somatodendritic 5-HT_1A_ receptors located on serotonin neuron cell bodies (Blier et al., 1998). Second, acute suppression of serotonin neuronal firing was mitigated by the acute accumulation of synaptic serotonin, via serotonin transporter protein blockade, thus maintaining 5-HT neurotransmission and continued stimulation of post-synaptic 5-HT_1A_ hippocampal receptors. Third, delay in antidepressant response was accounted for by a delay in down-modulation of somatodendritic 5-HT_1A_ autoreceptors following chronic serotonin agonism. Consideration of neuroplastic effects and intracellular transduction cascades (Duman et al., 1997, 1999, 2000) (Figure 1) following, for instance, post-synaptic agonism of hippocampal 5-HT_1A_ receptors (Radley and Jacobs, 2002; Santarelli et al., 2003; Fricker et al., 2005; Huang and Herbert, 2005, 2006) has been a later development of this model of SSRI efficacy. The Blier et al. SSRI model has remained ensconced while the field shifted to examine post-synaptic effects of serotonin neurotransmission including expression of early precursor gene products, expression of neurotrophic factors, and neurogenesis in the dentate gyrus of the hippocampus (Duman et al., 1997). However, salutary neurotrophic effects of SSRIs are rendered moot should serotonin neurotransmission be impeded, thus fundamentally undermining attempts to harness hippocampal neuroplasticity.

### Serotonin deficit hypothesis – human data

A leading hypothesis for SSRI efficacy is that depression results from a serotonin deficit state (Mann et al., 1996). However, studies measuring lumbar cerebrospinal fluid 5-hydroxy-indole-acetic acid (CSF 5-HIAA) in patients with major depression have failed to detect consistent reductions in CSF 5-HIAA in comparison to healthy volunteers (Bowers et al., 1969; Papeschi and McClure, 1971; Mendels and Frazer, 1974). One study using positron emission tomography examined cerebral blood flow patterns in response to fenfluramine, which induces release of presynaptic serotonin (Mann et al., 1996). The conclusions supported blunted regional cerebral blood flow responsivity in patients with major depression in comparison to controls (Mann et al., 1996), thus supporting the view of a regional serotonin deficit state in MDD. In addition, the inflammatory model of depression provides support for low serotonin neurotransmission inducing depression. Proinflammatory cytokines, such as interferon-γ, tumor necrosis factor-α, interleukin-6, and others, divert tryptophan from serotonin biosynthesis to quinolinic acid production through the kynurenic acid pathway, thereby decreasing serotonin production (Muller and Schwarz, 2007). In the latter scenario, a generalized serotonin deficiency is implied, but this view is complicated by other factors (see Serotonin and the Dorsal Raphe: The “Serotonin Flooding” Concept as Impediment to Serotonin Neurotransmission). The inflammatory model of depression, moreover, only applies to a subset of patients, raising the question why it has been so difficult to demonstrate a serotonin deficit state in “non-inflammatory” depression.

Our contention is that lumbar CSF represents the aggregate of excessive peri-raphe 5-HIAA and deficient synaptic serotonin at projection sites. Data from our laboratory support a “high peri-raphe 5-HT hypothesis” and thus model a condition for potential SSRI-treatment resistance (see Serotonin and the Dorsal Raphe: The “Serotonin Flooding” Concept as Impediment to Serotonin Neurotransmission). Using cisternal CSF taps in adversely reared non-human primates, we have noted *elevations* of cisternal CSF 5-HIAA in comparison to normally reared controls in two separate cohorts (Coplan et al., 1998; Mathew et al., 2002) and we have replicated the finding in a third cohort of differentially reared non-human primates (Fulton et al., 2014). When CSF is drawn via the cisternal route, which accesses the cerebellopontine angle, that CSF is expected to preferentially reflect diffusion from peri-raphe 5-HIAA. Lumbar CSF reflects, we argue, the aggregate of high raphe 5-HIAA and low fronto-limbic projection concentrations of 5-HIAA. This asymmetric distribution of serotonin – high midbrain and low prefrontal cortex 5-HT – is supported by findings in completed suicide victims (Bach et al., 2014). Thus, lumbar CSF 5-HIAA studies may *de facto* be a flawed method to document a serotonin deficit state since the aggregate fails to detect regional deficits. Rather, we endeavor to conceptualize TRD as an aberrant regional distribution of serotonin, with an ultimate reduction in serotonin neurotransmission at critical serotonin projection sites.

### Serotonin and the dorsal raphe: The “serotonin flooding” concept as impediment to serotonin neurotransmission

**Figure 2:**
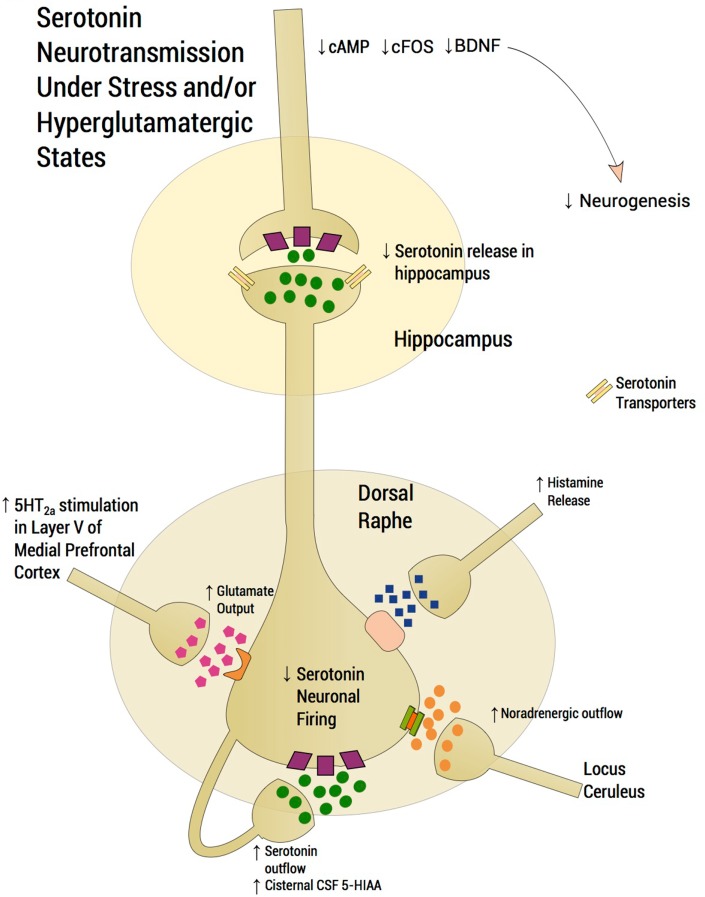
**Serotonergic neurotransmission under stress and/or hyperglutamatergic states**. Glutamatergic excitatory input activates a small population of serotonin neurons in the posterior part of the dorsal raphe, which leads to accumulation of extracellular serotonin, spreading to the anterior regions of the dorsal raphe thereby suppressing serotonin outflow through activation of somatodendritic 5-HT_1A_ autoreceptors. Stress induces glutamatergic neurotransmission directed into the dorsal raphe and stress therefore “floods” the dorsal raphe with serotonin, thus “choking” serotonin outflow. Activation of 5-HT_2A_ receptors in the mPFC activates Layer V cortical neurons enhancing glutamatergic input into the dorsal raphe. High peri-raphe 5-HT has been putatively demonstrated by high cisternal CSF taps in adversely reared non-human primates where elevations of CSF 5-HIA have been noted in adversely reared subjects in comparison to normally reared controls. Glutamate, serotonin, noradrenaline, and histamine are activated by stress and exert an inhibitory effect on serotonin outflow, in part, by “flooding” 5-HT_1A_ autoreceptors by serotonin itself. Factors, which may exacerbate this scenario, such as presence of the short arm of the serotonin transporter gene, early life adversity and comorbid bipolar disorder, are generally associated with SSRI-treatment resistance. An excess of midbrain peri-raphe serotonin is associated with a subsequent deficit at important fronto-limbic projection sites, with a compromise in serotonin-mediated neuroplasticity.

A number of preclinical studies have elucidated mechanisms by which the dorsal raphe serotonergic system is modulated. Medial prefrontal cortex (mPFC) glutamatergic projections to the dorsal raphe have been described in detail (Celada et al., 2001; Altieri et al., 2012). Thus, stimulation of the mPFC causes release of serotonin in the dorsal raphe via activation of glutamatergic-releasing pyramidal layer V neurons. The ensuing increase of extracellular serotonin in the dorsal raphe region inhibits serotonergic neuronal firing by acting on 5-HT_1A_ presynaptic autoreceptors thereby decreasing serotonin release in critical projection areas such as hippocampus and mPFC. Moreover, subsequent failure of activation of 5-HT_1A_ somatodendritic heteroreceptors on pyramidal cells in the mPFC further enhances glutamatergic outflow, exacerbating excessive serotonin release or what we have dubbed “serotonin flooding” in the vicinity of the dorsal raphe (Tao et al., 1997; Valentino et al., 2003; Altieri et al., 2012). Agonists activating NMDA and AMPA receptors on pyramidal cells in the mPFC mimic stress-related increases in glutamatergic neurotransmission. NMDA or AMPA receptor activation in mPFC further increases serotonin release in the dorsal raphe, with the expected activation of 5-HT_1A_ autoreceptors that decrease dorsal raphe serotonergic neuron firing. Thus, stress activates glutamatergic neurotransmission via NMDA or AMPA receptors in the mPFC (Musazzi et al., 2010) conceivably “flooding” the dorsal raphe with serotonin and attenuating serotonin outflow (Figure 2). According to the inflammatory hypothesis of depression, production of quinolinic acid, an NMDA agonist, is preferentially increased by proinflammatory cytokines. Thus, inflammation also activates glutamatergic neurotransmission, conceivably contributing to excessive 5-HT_1A_ autoreceptor agonism and an ensuing decrease in serotonin neuron firing (Muller and Schwarz, 2007). In this regard, we inform the readers that we may have selected references that are preferentially relevant to our hypothesis. By contrast, studies by Rozeske et al. (2011) reveal that uncontrollable rather than controllable stress leads to desensitization rather than downregulation of DRN 5-HT_1A_ receptors. This latter manuscript suggests that our hypothesis does not fully account for certain studies in the literature that are discrepant with our hypothesis.

### Role of 5-HT_1A_ receptor in depression and anxiety in animal models

In general, serotonergic neuronal firing is under tonic inhibition of somatodendritic 5-HT_1A_ autoreceptors, and their selective deletion causes an increase in extracellular serotonin in certain brain projection areas. New molecular techniques, such as RNA interference, which silences gene expression, are being used to detect the role of 5-HT_1A_ receptors in depression and anxiety. Collectively, these studies support the contention that attenuation of 5-HT_1A_ autoreceptor function enhances serotonin neuronal firing. Small interfering RNA (siRNA) contains about 25–50 bp of double stranded RNA, homologous to the gene to be silenced, by blocking transcription. Silencing the 5-HT_1A_ receptor gene by siRNA decreases 5-HT_1A_ receptor expression. In one study in mice, intracerebroventricular injection of siRNA targeting 5-HT_1A_ receptor mRNA, coupled with the SSRI sertraline, showed an acute antidepressant effect in the forced-swim and tail-suspension tests. When this conjugate was administered via intranasal route, effects similar to the intracerebroventricular route were observed, thereby opening new therapeutic strategies (Bortolozzi et al., 2012).

Outcomes of experiments with 5-HT_1A_ receptor knockout (KO) mice have observed to be dependent on many factors including whether the KO receptor is presynaptic (autoreceptor) or post-synaptic, and the timing of receptor suppression. It has been observed that lifelong deletion of 5-HT_1A_ receptors in mice predisposes to anxious behavior (Richardson-Jones et al., 2011). However, 5-HT_1A_ receptors play a role in neuronal development, such that complete KO of 5-HT_1A_ receptors from birth may well be associated with neurodevelopmental effects, which may influence behavioral outcomes. Inducible suppression of 5-HT_1A_ receptor provides a methodology of bypassing neurodevelopmental effects of lifetime KO (Richardson-Jones et al., 2010, 2011). Rodents with inducible 5-HT_1A_ autoreceptor suppression (low 5-HT_1A_ group) did not differ in behavioral response to conflict-based anxiety or an acute stressor in comparison to the high 5-HT_1A_ group. However, the former had increased mobility to a chronic stressor in the forced-swim test (Richardson-Jones et al., 2010). 5-HT_1A_ post-synaptic receptors have been found to be necessary for antidepressant effects; hence, 5-HT_1A_ autoreceptor selective antagonism coupled with post-synaptic 5-HT_1A_ receptor agonism is thought to produce antidepressant effects (see Bipolar Versus Unipolar Depression: Data from Animal Models). Thus, further evidence is provided that 5-HT_1A_ down-modulation plays a critical role in antidepressant response and has also been implicated in the expression of mood and anxiety-like behaviors (Richardson-Jones et al., 2010, 2011; Celada et al., 2013).

### Role of serotonin transporter in depression

**Figure 3:**
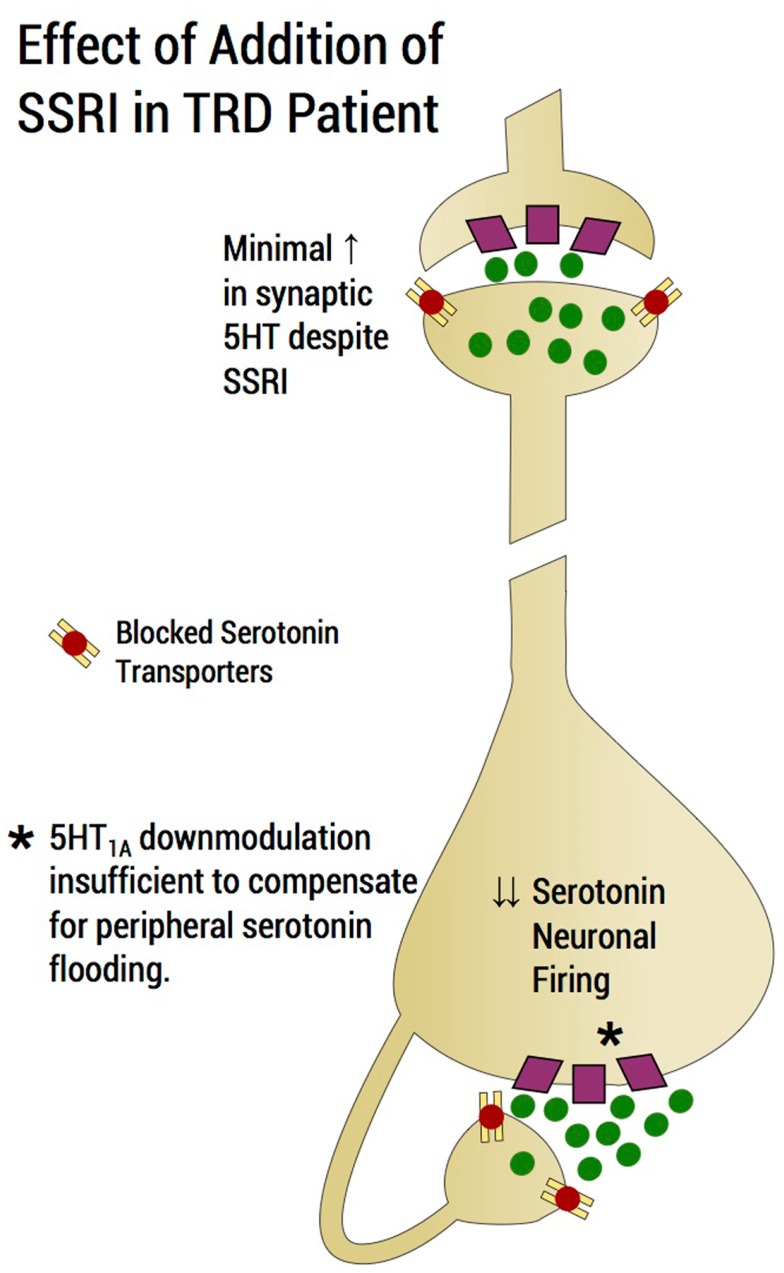
**Effect of addition of an SSRI in a TRD patient**. SSRI addition would induce a flood of serotonin in the region of the dorsal raphe and would tend to shut down serotonin neuronal firing through agonism of 5-HT_1A_ autoreceptors and would tend to exacerbate rather than improve a deficit state of serotonin at the distal projection site. Raising the dose of the SSRI, which is common practice in an upward titration strategy, may in fact, exacerbate the situation of flooding the extracellular fluid with serotonin and further preventing the possibility of serotonin neurotransmission enhancement. The serotonin system may impervious to the physiological process of an increment of SSRI producing a commensurate enhancement of serotonin neurotransmission. Rather, an increment in SSRI reduces the likelihood of serotonergic neurotransmission enhancement as the dosage increase exacerbates the build-up of serotonin in the peri-raphe area, thus enhancing shutdown of serotonin firing through 5-HT_1A_-autoreceptor agonism. Of note, the magnitude of peri-raphe serotonin build-up overwhelms the capacity of 5-HT_1A_ autoreceptor to down-regulate.

In humans, depression consequent to early-life stress is influenced by polymorphisms of specific genes that confer a vulnerability diathesis (Kaufman et al., 2004, 2006; Kendler et al., 2005; Popova Nina and Naumenko Vladimir, 2013). The serotonin transporter protein gene, SLC6A4, encodes the serotonin transporter, a protein critical to the regulation of brain serotonin function (Lesch et al., 1995). A serotonin transporter polymorphic site that maps to the promoter region, commonly known as 5-HTTLPR, consists of a variable number of tandem repeats. There are two common functionally different alleles at 5-HTTLPR, the short (“s”) allele and the long (“l”) allele. The “s” allele encodes an attenuated promoter segment, and, relative to the “l” allele, is associated with reduced transcription and functional capacity of the serotonin transporter (Lesch et al., 1995). The “l” allele can be subtyped into “La and Lg” alleles; the latter is thought to be similar to the “s” allele (Frodl et al., 2008). Studies have therefore examined the clinical influences of a triallelic (La-Lg-S) system (Stein et al., 2006). Although two meta-analyses refuted effects of the 5-HTTLPR on the relationship between concurrent stress and depression (Munafo et al., 2009; Risch et al., 2009), these have been criticized (Rutter et al., 2009; Uher and McGuffin, 2010). In addition, a more recent larger meta-analysis supported the hypothesis that 5-HTTLPR is involved in the interaction between stress and depression (Karg et al., 2011). Superior response to SSRIs in depressed patients with the long allele versus short allele has been reported (Smits et al., 2008; Gressier et al., 2009; Huezo-Diaz et al., 2009; Min et al., 2009; Illi et al., 2011), although some studies fail to show allelic effects (Lewis et al., 2011). Although description of the functional attributes of the serotonin polymorphism have been contradictory (Karg et al., 2011), clinical data would prompt us to suspect that the short allele is associated with an inability of the serotonin transporter to cope with excess synaptic 5-HT in the somatodendritic raphe area but this statement remains speculative. Therefore, compromised SSRI response in patients with the short allele potentially invokes an autoreceptor mechanism, in that short allele subjects are likely to accumulate serotonin in the peri-raphe area. Further implicating the 5-HT_1A_ autoreceptor and the 5-HTTLPR in SSRI response, antidepressant effects were boosted by the 5-HT_1A_ antagonist, pindolol, specifically in MDD patients with the short allele serotonin transporter genotype (see below in this section) (Zanardi et al., 2001). Similarly, poor SSRI response in patients possessing the short allele of the 5-HTTLPR is observed in generalized social anxiety disorder (Stein et al., 2006) and in panic disorder in female patients (Perna et al., 2005). SSRIs may conceivably exacerbate a condition of insufficient peri-raphe clearing of serotonin in subjects with the short allele serotonin transporter gene. The expected compensation of autoreceptor down-modulation to enhanced peri-raphe 5-HT is overwhelmed by the magnitude of agonism during “flooding” and putatively exceeds the capacity of the autoreceptor to sufficiently “down-modulate” (Blier et al., 1998) (Figure 3), a contention supported by 5-HT_1A_ irreversible KO rodent studies described below (see Bipolar Versus Unipolar Depression: Data from Animal Models). We speculate that with inadequate 5-HT_1A_-autoreceptor down-modulation, restoration of dorsal raphe firing following SSRI administration cannot be assured.

### Role of 5-HT_1A_ genes in depression

With regards to the 5-HT_1A_ receptor gene, transcriptional modifications have been observed in depression (Celada et al., 2013). Factors such as glucocorticoids, brain-derived neurotrophic factor (BDNF), and proteins such as deformed epidermal autoregulatory factor-1 (Deaf-1), Freud-1, Hes 5, and Hes 1 have been associated with transcriptional regulation of the 5-HT_1A_ gene (Celada et al., 2013). Functional polymorphisms in the promoter region of the Htr_1A_ gene (which codes for 5-HT_1A_ receptors) are associated with variable response to antidepressants. In mice studies, lack of the Deaf-1 protein was found to be associated with increased presynaptic 5-HT_1A_ receptor function/expression and decreased 5-HT_1A_ receptor post-synaptic function/expression (Celada et al., 2013). Freud-1 is a transcriptional protein that is thought to play a role in autoregulation of the serotonin system. It has been observed that chronic activation of 5-HT_1A_ receptors leads to increased expression of Freud-1, which causes decrease in 5-HT_1A_ receptor gene expression (Popova Nina and Naumenko Vladimir, 2013). Presence of the C(-1019)G polymorphism in the 5-HT_1A_ gene promoter region, which prevents 5-HT_1A_ gene repression, results in higher 5-HT_1A_ receptor expression, explaining failure of antidepressant response (Celada et al., 2013). Thus, SSRIs may conceivably exacerbate a reduction in serotonin neuronal firing in persons with the C(-1019)G polymorphism of the 5-HT_1A_ gene because of 5-HT_1A_ autoreceptor overexpression.

### Perils of SSRI dosage increase to pre-existing peri-raphe serotonin

After SSRI initiation, as mentioned previously, an initial elevation of extracellular serotonin in the peri-raphe region results in decreased serotonergic firing via 5-HT_1A_ autoreceptor agonism. This is compensated for by the elevated synaptic serotonin at post-synaptic sites through transporter blockade at the distal presynaptic site. For an antidepressant response to occur, sufficient down-modulation of 5-HT_1A_ autoreceptors is required to allow for recovery of firing of the serotonin neurons (Bose et al., 2011). Raising the SSRI dose in our model of the TRD patient would putatively exacerbate flooding of peri-raphe extracellular fluid with serotonin and prevent enhancement of neurotransmission, leading to non-response (Figure 3). The serotonin system becomes, under these circumstances, impervious to SSRI dosage increase, failing to produce the desired enhancement of serotonin neurotransmission. By contrast, in such patients, SSRI increments are speculated to reduce the likelihood enhancing of serotonin neurotransmission as dosage increases conceivably exacerbate the build-up of serotonin in the peri-raphe area, thereby producing additional shutdown of serotonin firing through 5-HT_1A_ autoreceptor agonism (Figure 3). In the STAR*D, SSRI non-response appears not attributable to the type of SSRI administered. Switching to a second SSRI, only achieved a remission rate of <20% (Rush et al., 2006), suggesting that factors, which rendered the patient impervious to the first SSRI, largely persisted for the second SSRI trial. The corollary is that non-response to a single SSRI is predictive of continued resistance to SSRIs (Rush et al., 2006). Sustained non-response to escalating SSRI doses is the first indicator of “failing ‘a putative’ SSRI challenge test” (Table 1).

**Table 1:** **Failure of the “SSRI challenge” test-clinical features**.

Antidepressant-induced mania
Jitteriness syndrome
Tachyphylaxis
Non-response

*The phenomenon of non-response to escalating SSRI doses is the first indicator of “failure” of a putative “SSRI challenge test.” Other indicators of a failed SSRI challenge test are exhibiting mania or hypomania, tachyphylaxis (often accompanied by rapid response), and/or paradoxical response to SSRIs. Such individuals are predicted to respond poorly to future SSRI trials*.

Alternatively, 5-HT_1A_ autoreceptor antagonism in conjunction with serotonin transporter reuptake blockade could theoretically produce rapid antidepressant effects and alleviate the effects of “serotonin flooding.” A systematic review of the literature involving augmentation of SSRIs by pindolol, a 5-HT_1A_ antagonist, reveals rapid antidepressant effects which were evident within 2 weeks of pindolol augmentation in certain studies. But the area has yielded conflicting results (Whale et al., 2010). This ambiguity may, in part, be due to the non-selective full blockade of pre- and post-synaptic 5-HT_1A_ receptors by pindolol, post-synaptic 5-HT_1A_ receptor activation being necessary for preserving antidepressant effects. Moreover, it was not clear during the initial pindolol studies that serotonin transporter gene polymorphisms play a critical role in SSRI response. The new antidepressant, vilazodone, was developed with a similar concept in mind and has combined 5-HT_1A_ partial agonistic activity and serotonin transporter reuptake blockade (Hughes et al., 2005). The crucial difference between vilazodone versus pindolol augmentation is that the latter is a full antagonist whereas the former is a potent partial agonist (Hughes et al., 2005; Khan, 2009), as is aripiprazole (Stahl, 2001) thus permitting activation of post-synaptic 5-HT_1A_ receptors (discussed further in Section “A Psychopharmacological Approach to Treatment-Resistant Depression”).

### Basis for serotonin reuptake enhancement as antidepressant

Tianeptine (Stablon) is used for the treatment of MDD in Europe but not in the US (Lejeune et al., 1988). Tianeptine’s mechanism of action ostensibly defies logic as it enhances rather than inhibits reuptake of serotonin at the serotonin transporter site. Human studies show rapid relief of depressive symptoms (Novotny and Faltus, 2003) and good tolerability of tianeptine as compared to SSRIs (Atmaca et al., 2003; Bonierbale et al., 2003). One possible mechanism of action of tianeptine is that it blocks stress-induced glutamate transporter-1 mRNA (GLT-1 mRNA) expression and stress-induced decreases in hippocampal volume (Reagan et al., 2004). The hypothesis of GLT-1mRNA inhibition underlying the mechanism of action of tianeptine could be integrated with our above stated contention. Glutamatergic activation of pyramidal cells causes increase of dorsal raphe extracellular serotonin. Hence inhibiting GLT-1mRNA prevents accumulation of dorsal raphe extracellular serotonin.

Another explanation that may complement tianeptine’s therapeutic mechanism of action is that it is may be best suited for depression associated with high peri-raphe serotonin. By presumably clearing the peri-raphe area of 5-HT, the 5-HT_1A_-somatodendritic autoreceptor is vacated, and the dorsal raphe neuron can now resume firing. However, the somatodendritic effect is putatively offset by enhanced synaptic reuptake of serotonin at projection sites, conceivably depriving post-synaptic 5-HT_1A_ receptors of serotonin. A PET study by our group, using the McNeil [^11^C McN 5652] compound, which labels serotonin transporter, shows numerically higher concentrations of serotonin transporter in the raphe area vis-à-vis hippocampus (Kent et al., 2002), thus raising the possibility that it is tianeptine’s peri-raphe effects that are most germane to its serotonergic efficacy. Certainly, in animal models of chronic restraint stress, tianeptine was effective whereas SSRIs did not reverse stress-induced behavioral and biological changes (McEwen et al., 2009).

## Untoward Effects of “SSRI Challenge”

Clinical features evident during medication trials may conceivably predict future SSRI non-response. Information may be gleaned from observations made during what we have dubbed the “SSRI challenge” test (Table 1). Thus, a patient may “fail” the “challenge” imposed by SSRI administration.

### Antidepressant-induced mania

The first, most obvious, failed outcome is when an SSRI is prescribed for purported unipolar depression and induces mania, hypomania, rapid cycling, or mixed states. A prior history of bipolar mood disorder is often elicited upon more thorough inquiry. This form of bipolarity has previously been termed Bipolar III (Akiskal and Pinto, 1999) stating “a full hypomanic episode emerging during antidepressant treatment and persisting beyond the physiological effect of that treatment” (Chun and Dunner, 2004; Fiedorowicz et al., 2011). Adolescents who have not manifested the manic component of a bipolar disorder may be particularly vulnerable to SSRI antidepressant-induced mania or hypomania (Frye et al., 2009). Although preliminary studies showed conflicting results regarding the association between 5-HTTLPR polymorphism and antidepressant-induced mania (Mundo et al., 2001; Rousseva et al., 2003; Serretti et al., 2004; Baumer et al., 2006; Masoliver et al., 2006; Ferreira Ade et al., 2009), a recent meta-analysis indicated a higher incidence of antidepressant-induced mania in patients with the “s” variant of 5-HTTLPR (Daray et al., 2010). Likelihood of SSRI monotherapy response is deemed low once failure of the “SSRI challenge” test has been observed in this category. Further discussion regarding unipolar versus bipolar depression in the context of TRD is discussed below (see Bipolar Versus Unipolar Depression).

In TRD, an SSRI may conceivably induce hypomania or mania by excessively increasing distal serotonin outflow because the “brake” system, which is comprised of 5-HT_1A_ autoreceptors, is downmodulated following chronic agonism. This hypothesis is supported by PET imaging (Drevets et al., 1999), which shows that a familial form of unipolar disorder exhibit reduced ligand binding of raphe 5-HT_1A_ receptors. A subgroup with the lowest 5-HT_1A_ binding had a positive family history of bipolar disorder, which may represent a subthreshold phenotype of bipolar disorder (Drevets et al., 1999). Thus, precedent exists in the clinical literature for down-modulation of the 5-HT_1A_ “brake” system specifically in those individuals where we have contended SSRIs are likely to be ineffective.

### Jitteriness syndrome

In the second instance, the patient develops a “paradoxical” reaction to the SSRI, which may include “the jitteriness syndrome” (Gorman et al., 1987). The paradoxical reaction consists of marked sensitivity to SSRIs with development of increased anxiety, jitteriness, or a feeling of “jumping out one’s skin” and worsening depression and irritability. The paradoxical reaction may be responsible for the emergence of suicidal ideation in the context of SSRI administration (Hammad et al., 2006; Perlis et al., 2007). The occurrence of a paradoxical reaction to an SSRI may not bode well for future SSRI-treatments because of SSRI intolerance. In panic disorder, recommendations were to initiate treatment at low doses with gradual titration. However, a similar reaction may occur in patients with MDD even without panic disorder (Beasley et al., 1991, 1992). It was speculated that the reaction occurred from “upregulated” 5-HT_2_ receptors with abatement of the “jitteriness” syndrome and clinical response coinciding with the down-modulation of 5-HT_2_ receptors (Gorman et al., 1987). Alternatively, the jitteriness syndrome may represent an acute shutdown of dorsal raphe firing to an extent that compromises distal synaptic cleft serotonin following serotonin reuptake inhibition. Perlis et al. (2003) report that the jitteriness syndrome was associated with excess rates of the “s” allele in accordance with the notion that there is a build-up of peri-raphe serotonin when transporter reuptake is sluggish and serotonin neurotransmission is then impeded. This type of clinical presentation is also deemed to represent a failure of the SSRI “challenge” test.

### Tachyphylaxis and rapid response

The third scenario for failure of the SSRI challenge test is tachyphylaxis, or what has commonly been generically termed “poop out,” in response to an SSRI (Trivedi et al., 2008). Katz reported high rates of tachyphylaxis on SSRIs when compared to other antidepressants, in patients with dysthymia (Katz, 2011). Similarly, a study by Akiskal et al. (2006) reports a high incidence of hyperthymia and hypomania following antidepressant therapy. The literature describes the existence of a possible “depressive inhibition” in bipolar disorder that “overshoots” into a hypomanic state (Himmelhoch, 1998). Despite an extensive literature search, we have not identified references for the co-occurrence of hyperthymia and ensuing tachyphylaxis. Anecdotally, many patients who experience tachyphylaxis are found to have experienced an initial period of rapid onset hyperthymia occurring within days following SSRI initiation. This latter phenomenon is a topic that requires further research. Rapid and dramatic response to an SSRI does not usually bode well for durability. Quitkin et al. (1998) indicated that “placebo” response was often accompanied by an acute, remitting-relapsing pattern, whereas true drug response was associated with slow and persistent response. Bupropion has been anecdotally favored for addition to SSRIs to regain treatment response (Posternak and Zimmerman, 2005). It is our contention that tachyphylaxis indicates a fundamental inability of serotonin mechanisms to maintain treatment response, although the mechanisms for the collapse of serotonin outflow remain unclear. Usually augmentation strategies, described below in the Section “A Psychopharmacological Approach to Treatment-Resistant Depression,” are required.

### Minimal response

The fourth scenario is when the patient experiences very little if any response to an adequate SSRI trial, indicating that attempting to boost distal synaptic serotonin has failed as a strategy. In many instances, the non-response has been observed several times, as was the case in the STAR*D (Rush et al., 2006). We have already provided a biological model of serial SSRI-treatment resistance based on glutamatergic-induced flooding of serotonin activating the 5-HT_1A_ autoreceptors (Valentino et al., 2003; Amat et al., 2005; Altieri et al., 2012). Activation of 5-HT_2A_ receptors in the mPFC activates layer V cortical neurons whereas activation of 5-HT_1A_ receptors diminish glutamatergic outflow from the mPFC. Thus, 5-HT_2A_-receptor antagonism in the mPFC, a receptor-binding feature common to all atypical antipsychotics, conceivably enhances serotonin outflow by blocking glutamatergic input into the dorsal raphe. In addition, 5-HT_1A_ partial agonism, an effect of certain atypical agents, may mediate a distal inhibition of glutamatergic input into the dorsal raphe, thus retaining integrity of serotonin activation through other afferent sites, such as the lateral habenula (Aghajanian and Wang, 1977; Stern et al., 1981; Kalen and Wiklund, 1989), noradrenergic nuclei, substantia nigra, and the spontaneous firing rate intrinsic to the dorsal raphe neurons (Celada et al., 2001). In summary, activation of 5-HT_2A_ receptors in the mPFC is associated with increased glutamatergic tone in the dorsal raphe; a reduction of serotonin outflow and therapeutic effects of SSRIs will be overwhelmed. Taken together, “flooding” of the dorsal raphe 5-HT_1A_-autoreceptors by serotonin surplus is hypothesized to lead to certain instances of SSRI-treatment resistance. We therefore complete the four scenarios depicting failure to the SSRI challenge test that should alert the clinician that success with SSRI agents is unlikely.

## Bipolar Versus Unipolar Depression

As described in these four scenarios, the extent to which treatment-resistant depression overlap with variants of bipolar spectrum disorder may be great. Studies reveal that the misdiagnosis of bipolar disorder is in the range of approximately 70% and the majority of those misdiagnosed receive a diagnosis of MDD (Hirschfeld et al., 2003). It has been well documented that patients with bipolar disorder respond poorly to antidepressants (Sachs et al., 2007). To what extent, therefore, are TRD samples contaminated by covert or overt bipolarity? While a recent report showed a nearly 40% rate of subthreshold hypomania in a group with MDD (Angst et al., 2010), another study (Perlis et al., 2011) indicated that undiagnosed bipolar spectrum disorder did not substantially contribute to non-response in STAR*D. A comprehensive meta-analysis (Correa et al., 2010) addressing the issue of whether unrecognized bipolar disorder is a significant contributor to apparent treatment resistance was deemed to receive moderate support. Less than moderate support was evinced for the view that relapses during ongoing antidepressant treatment (tachyphylaxis) are likely to have bipolar spectrum disorder although few of the studies reviewed were specifically designed to address this question. Also, moderate support was provided for the view that antidepressants were not “robustly” effective in the face of a bipolar disorder diagnosis.

The neurobiology of the switch into mania has been discussed by other investigators (Salvadore et al., 2010). In the current model, the critical “switch” into mania or hypomania occurs when glutamatergic “inhibition” of the dorsal raphe is no longer active or the mPFC no longer provides glutamatergic excitation from its layer V pyramidal neurons, although the reason for glutamatergic quiescence remains unclear. The dorsal raphe is now, we posit, relatively free to “overshoot” as the brake system dependent on the sensitivity of the 5-HT_1A_ autoreceptors has been compromised by chronic agonism. An excess of serotonin may inundate distal synaptic sites, which includes the nucleus accumbens (Millan et al., 2000) where excessive hedonic responses are likely to be elaborated. Below, in the animal model section, we examine the plausibility of the aforementioned scenario in mice with selective 5-HT_1A_ autoreceptor KO.

### Bipolar versus unipolar depression: Data from animal models

Rodent models are argued to be relevant to the occurrence of mania, hypomania, or bipolar type III, specifically in the context of TRD (Holmes et al., 2003; Ansorge et al., 2004). The first model entails the study of adult KO mice lacking the gene for, and therefore lifelong cessation of serotonin transporter protein expression (mentioned previously in Section “The Role of 5-HT_1A_ Receptor in Depression and Anxiety in Animal Models”) (Holmes et al., 2003). These KO mice purportedly mimic humans monozygotic for the short arm of the serotonin transporter gene. Concentration of peri-raphe serotonin is high in comparison to wild-type mice and dorsal raphe serotonin neurons are in a perpetual state of hyperpolarization (Lira et al., 2003). Distal synaptic serotonin concentrations at projection sites such as the hippocampus are, as predicted, greatly diminished (Holmes et al., 2003). Moreover, there is a marked down-modulation of 5-HT_1A_ autoreceptors in the dorsal raphe in response to the chronic agonist activity of serotonin, but not of sufficient magnitude to sustain normative rates of serotonin neuronal firing. Thus, there is an upregulation of post-synaptic 5-HT_1A_ receptors in the hippocampus reflecting the low concentrations of serotonin at the distal synapse. Once a serotonin 5-HT_1A_ antagonist, such as WAY 100635, is applied to dorsal raphe neurons, there is a dramatic increase in dorsal raphe neuronal firing rates since the antagonist preferentially interrupts the hyperactive negative feedback system, eliciting a rebound overshoot (Holmes et al., 2003). The serotonin “overshoot” is argued to mimic serotonin neurotransmission in manic or hypomanic states in humans, although what the physiological parallel to 5-HT_1A_ antagonism is remains unclear. A similar scenario has been observed following exposure of rodents to SSRIs early in life (Ansorge et al., 2004). Such rodents resemble serotonin transporter KO mice with high peri-raphe serotonin and down-modulation of 5-HT_1A_ autoreceptors (Ansorge et al., 2004). The latter data imply that SSRIs administered developmentally may primarily function in a similar fashion as an irreversible serotonin transporter KO mouse. By contrast, inducible selective KO of 5-HT_1A_ autoreceptors in mice causes an increase in extracellular serotonin in the PFC but not in the hippocampus at baseline (Richardson-Jones et al., 2011). There was, however, a significant increase in extracellular serotonin in the hippocampus of autoreceptor KO mice when an SSRI was administered, while no increase of extracellular serotonin was found in the PFC following SSRI administration.

## Psychopharmacological Approach to Treatment-Resistant Depression (Figure 4)

Based on the current proposal, we are therefore guided toward a rational psychopharmacological approach to TRD, which is deemed relevant to a majority of patients encountered in the STAR*D study. Drugs with a diverse repertoire of mechanisms of action may be rationally combined to enhance sluggish serotonin neurotransmission (Millan, 2013). Certainly, polypharmacy with antipsychotics has been debated for treatment of schizophrenia and no clear guidelines exists (Stahl, 2002). A fundamental basis for the polypharmacy regimen proposed below is administration of an SSRI as the “augmentation substrate,” despite the likelihood that patients with TRD will not remit or respond to an SSRI. Certainly, there are exceptions. For instance, lamotrigine has been shown effective as monotherapy for the prophylaxis of mood episodes in Bipolar I patients, both for manic and depressive recurrence. There is evidence that lamotrigine was more effective for the prophylaxis of depressive episodes while lithium was more effective for prophylaxis of manic episodes (Calabrese et al., 2003; Goodwin et al., 2004). Moreover, quetiapine and lurasidone have been FDA approved as monotherapy for bipolar depression (Calabrese et al., 2005; Thase et al., 2006; Yatham et al., 2013) and quetiapine monotherapy was as effective as duloxetine for MDD (Cutler et al., 2009; McIntyre et al., 2009). However, the combination of olanzapine and fluoxetine was more effective for TRD than either drug alone, supporting the utility of an SSRI as an augmentation substrate (Corya et al., 2006).

**Figure 4:**
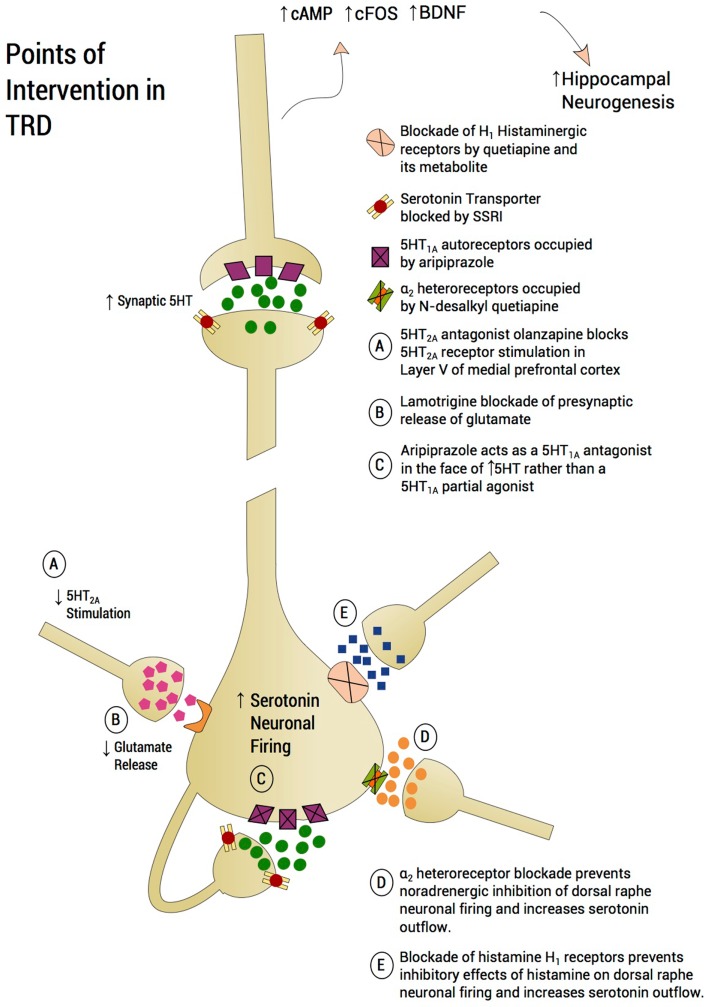
**Points of intervention in TRD**. A treatment approach on a plausible neurobiological model is proposed, which aims to prevent mPFC-induced serotonin shutting down the dorsal raphe. Utilizing an incremental approach, lamotrigine is recommended as a first step to curb glutamatergic inflow to the dorsal raphe. Aripiprazole will further reduce raphe glutamatergic input by blocking 5-HT_2A_ receptors in the medial prefrontal cortex and protecting 5-HT_1A_ autoreceptors from serotonin auto-inhibition through partial 5-HT_1A_ agonism. Quetiapine’s metabolite, *N*-desalkyl quetiapine, acts as an antagonist at α_2_-heteroreceptors, thus protecting the raphe neurons from shutdown by norepinephrine overdrive. Finally, drugs like quetiapine also possess potent antihistaminergic effects for histamine H_1_ receptors. Histamine through H_1_ may reduce serotonin firing. Based on the view that each neurotransmitter system mentioned – glutamate, serotonin, noradrenaline, and histamine – are activated by stress and exert a partial inhibitory effect on serotonin outflow, monotherapy may well be inadequate. Rather, a “stacking” approach to pharmacotherapy is proposed and creating a formal database using this method is worthy of pursuing.

The first augmentation agent that we recommend is lamotrigine. One disadvantage is the time required to raise the lamotrigine dosage. Although a number of lamotrigine augmentation studies in unipolar depression showed significant antidepressant effect (Rocha and Hara, 2003; Gutierrez et al., 2005; Gabriel, 2006), especially in severely treatment-resistant patients (Barbee and Jamhour, 2002), one study reported negative results (Santos et al., 2008). Moreover, a recent placebo-controlled study failed to show significant difference between lamotrigine and placebo as augmentation to paroxetine in unipolar depression (Barbee et al., 2011). However, two findings reported by this study are consistent with our recommendation of lamotrigine as the first augmentation agent in a well-characterized TRD group. First, the study showed that the antidepressant effect of lamotrigine is more prominent in treatment-resistant patients, in line with our targeted group. Second, the data suggested a placebo and lamotrigine separation by the last visit with completers of 10 weeks treatment approaching significance, an observation that was attributed to the required slow titration of lamotrigine (Barbee et al., 2011). Taken together, these data suggest a significant antidepressant effect for lamotrigine augmentation but only when used for a longer period of time and in TRD patients.

Patients with bipolar “spectrum” disorder, “soft” bipolar disorder, or “pseudounipolar” depression (Ghaemi et al., 2000) appear to respond particularly well to lamotrigine with an SSRI, although formal studies are lacking. Referring back to our model, a critical agenda to restoring serotonin outflow is to reduce glutamatergic input into the raphe arising from pyramidal cells in layer V of the mPFC. By acting on sodium and calcium ion channels, lamotrigine inhibits presynaptic release of glutamate (Lees and Leach, 1993), and thus prevents excessive stimulation of serotonin, which secondarily would impede the shutdown of dorsal raphe serotonin neuron firing. Two other glutamate modulating agents have been successfully used in TRD as monotherapy: (1) riluzole, an FDA approved for amyotrophic lateral sclerosis, with a complex mechanism of action including glutamate release inhibition and reuptake enhancement (Zarate et al., 2004, 2005; Sanacora et al., 2007), and (2) sub-anesthetic doses of ketamine infusion, a dissociative anesthetic agent that possesses NMDA antagonist effects (Berman et al., 2000; Zarate et al., 2006; Valentine et al., 2011). Relevance of the antiglutamatergic effects of lamotrigine is suggested by the rapid onset of ketamine, also an antiglutamatergic through inhibition of NMDA receptors. In the latter case, blockade of excessive glutamatergic input to the DRN is postulated to occur rapidly. It remains unstudied to what extent, if at all, serotonin neurotransmission plays a role in the synaptogenesis and immunomodulation that underlies ketamine’s antidepressant properties, as reported by Zunszain et al. (2013). In the case of ketamine, it has been postulated that there is an increase of AMPA/NMDA neurotransmission, which is thought to cause an increase in dendritic spine formation in the prefrontal cortex (Zunszain et al., 2013). Activation by ketamine of the serine/threonine kinase enzyme, the mammalian target of rapamycin (mTOR), is reported to cause the observed increase in synaptogenesis in the prefrontal cortex (Dwyer et al., 2012). The rapid mTOR mechanism specifically in layer 5 of mPFC entails dendritic spine increases and may well affect the pyramidal neurons that constitute afferents to the DRN (Li et al., 2010). It could also, however, be posited that ketamine effects are mediated downstream to serotonin modulation. Therefore, ketamine’s mechanism of action is less affected by the putative upstream serotonin shutdown. Another caveat is that ketamine’s pharmacological effect is short but the antidepressant effect lasts for days, presumably in the absence of NMDA antagonism. Also, sub-anesthetic doses, of ketamine increase (not decrease) mPFC glutamatergic output. Therefore it is conceivable, yet unstudied, that the local dorsal raphe increases in serotonin are NMDA dependent and could be blocked by ketamine. In summary, the extent to which serotonin neurotransmission plays a role in ketamine’s stimulation of synaptogenesis and immunomodulation remains unstudied.

The next step we recommend is addition of aripiprazole to the pre-existing regimen. We refer to the addition of successive augmenting strategies as “stacking,” rather than using one augmentation agent at a time. Stacking or polypharmacy has been essential for medical conditions such as diabetes and treatment-resistant hypertension to achieve clinical response by targeting disparate systems with similar physiological effects (Munger, 2010). The combination of drugs with divergent mechanisms of action and targeting of serotonin neurotransmission through diverse mechanisms may similarly achieve better outcomes in TRD, with the potential for reduced cumulative dosing of each medication used and minimizing side effect profiles (Munger, 2010). There may be multiple points at which the serotonin system may be compromised in TRD and in some individuals, it may be incumbent to address each of these inhibitory inputs until serotonin outflow is fully restored and clinical response, if not remission, is attained. Adding aripiprazole further attempts to impede exuberant negative feedback at the 5-HT_1A_ autoreceptors by possessing a potent 5-HT_1A_ partial agonist (Jordan et al., 2002). Since the peri-raphe concentration of serotonin is posited to be high, aripiprazole is predicted to act more as a full 5-HT_1A_ antagonist rather than a partial 5-HT_1A_ agonist, producing a rebound surge in serotonin outflow (Artigas et al., 2006) putatively aided by the reduction of glutamatergic outflow by lamotrigine. The FDA approval of aripiprazole as an augmenting agent for partially responsive depression separated from placebo by 1 week (Berman et al., 2009) suggesting that the mechanism of action was not delayed by the necessity for 5-HT_1A_ autoreceptor down-modulation. No formal studies we are aware of have been conducted combining an SSRI, lamotrigine, and aripiprazole. There is, however, a neurobiological rationale for the “stacking” of these two medications with an SSRI by virtue of utilizing two distinct mechanisms for preventing serotonin neurotransmission from shutdown following excessive negative feedback.

A question arises regarding the comparability of utility of aripiprazole, an atypical antipsychotic with 5-HT_1A_ partial agonist properties, combined with an SSRI versus the novel antidepressant, vilazodone (Viibryd), whose mechanism is described above in Section “Perils of SSRI Dosage Increase to Pre-Existing Peri-Raphe Serotonin” (Hughes et al., 2005; Khan, 2009). We would predict that by preventing early phase (<2 weeks) SSRI-mediated inhibition of dorsal raphe neuronal firing, vilazodone would be accompanied by a rapid antidepressant response. One study did indeed show a separation of drug from placebo by 1 week (Khan et al., 2011). The second pivotal trial failed to replicate the rapid onset of efficacy (Reed et al., 2012). Additional studies are therefore required. Comparative trials between vilazodone and other SSRIs or the use of vilazodone in TRD patients have not, to our knowledge, been performed. However, there is a possibility that aripiprazole also contributes to diminished glutamatergic outflow at the dorsal raphe by antagonizing 5-HT_2A_ receptors on layer V pyramidal neurons (Celada et al., 2001), thus additionally contributing to the restoration of serotonin neurotransmission. Thus, the addition of atypical antipsychotics in general, such as olanzapine, promotes 5-HT_2A_ receptors antagonism, which may represent an effective proximal mechanism for attenuating glutamatergic outflow to the dorsal raphe.

When the patient has yet to respond adequately, targeting the histamine system is proposed. Histamine, a monoamine, is increased by acute stress (Ito, 2000) and also increases the rodent stress response (Cote and Yasumura, 1975). Histamine exerts an inhibitory effect on serotonin neuronal firing through histamine H_1_ receptors (Brown et al., 2002). Quetiapine, and in particular its primary metabolite, *N*-desalkyl quetiapine (NQuet), are very potent inhibitors of the H_1_ receptor (Nikisch et al., 2010), and may thus facilitate an increase in serotonin neurotransmission via histaminergic antagonism *per se* (McIntyre et al., 2007). Rodent studies of quetiapine and NQuet, a potent norepinephrine reuptake inhibitor, indicate that the combination of quetiapine with or without an SSRI potently increases 5-HT neuronal firing through two mechanisms; via antagonism of α_2_-heteroreceptors located on serotonin neurons by NQuet and possibly via direct 5-HT_1A_ agonism by NQuet (Chernoloz et al., 2012). Since many patients with bipolar depression and/or TRD have previously failed trials with serotonin–norepinephrine reuptake inhibitors, there may be an important role for α_2_ heteroreceptor antagonism in quetiapine’s action. However, the potent antihistaminergic effects of NQuet may also contribute to an increase in serotonin neurotransmission (Dell’Osso et al., 2011).

Inhibitory influences on dorsal raphe firing may represent individual effects of activation of multiple neurotransmitters. In order to attain full remission, and remove a maximum of inhibitory effects, “stacking” of pharmacological agents may be necessary. If a particular agent is found to be inadequately effective but is discontinued before adding the next agent, the next agent has to “make up the ground” of the now unattended receptor/neurotransmitter system. “Stacking” is an approach used for treatment of hypertension (Munger, 2010) but is not advocated for in TRD, a disorder that may be argued is just as severe, incapacitating and difficult to treat as hypertension.

There are evidence-using meta-analyses that SNRI’s may possess superior efficacy to SSRIs when remission is used as the outcome variable (Thase et al., 2001). By blocking norepinephrine reuptake, norepinephrine outflow is diminished through agonism of α_2_ autoreceptors, which, in turn, diminishes inhibition of dorsal raphe serotonin neuron firing by decreasing norepinephrine neurotransmission through α_2_-heteroreceptors. Tricyclic antidepressants are also potent norepinephrine reuptake inhibitors and may also be expected to increase serotonin outflow (Charney et al., 1981), although the class is also credited with sensitizing post-synaptic 5-HT_1A_ receptors (Blier et al., 1987). Certainly, in our model of serotonin shutdown there is a cogent argument for reducing the inhibitory effects of norepinephrine on serotonin neuron firing.

## Conclusion

Animal models conceivably elucidate why SSRI-treatment for depression may underperform in patients with MDD. To summarize, an excess of serotonin is present at 5-HT_1A_ autoreceptors and a deficit exists at the post-synaptic sites, where the neurotransmitter is required for neurotrophic effects. Certain circumstances worsen the scenario, including early-life adversity, possession of the short arm of the serotonin transporter gene polymorphism and presence of bipolar disorder or bipolar spectrum disorder. We provisionally predict who will perform poorly on SSRIs, as they will have failed the SSRI challenge test by either exhibiting: mania or hypomania, tachyphylaxis, paradoxical response, or non-response. The treatment approach is based on a plausible neurobiological model, which aims to prevent excess serotonin shutting down the dorsal raphe. A “stacking” approach to pharmacotherapy is proposed although creating a formal database is a formidable task, yet one worthy of pursuit. Finally, we provide the caveat that the current manuscript is speculative in parts, based on clinical observation but supported by translational neuroscience. We posit that the manuscript may act as a catalyst for formal testing of proposed hypotheses and inform treatment of the TRD patient.

## Conflict of Interest Statement

Jeremy D. Coplan is on the Pfizer and Corcept advisory board and has given talks for Sunovion, Forest, Otsuka, BMS, AstraZeneca, GSK, Forest, Novartis and Pfizer. No biomedical financial interests or potential conflicts of interest are reported for Srinath Gopinath, Chadi G. Abdallah, Benjamin R. Berry.
